# Analysis of the Salivary Gland Transcriptome of Unfed and Partially Fed *Amblyomma sculptum* Ticks and Descriptive Proteome of the Saliva

**DOI:** 10.3389/fcimb.2017.00476

**Published:** 2017-11-21

**Authors:** Eliane Esteves, Sandra R. Maruyama, Rebeca Kawahara, André Fujita, Larissa A. Martins, Adne A. Righi, Francisco B. Costa, Giuseppe Palmisano, Marcelo B. Labruna, Anderson Sá-Nunes, José M. C. Ribeiro, Andréa C. Fogaça

**Affiliations:** ^1^Departamento de Imunologia, Instituto de Ciências Biomédicas, Universidade de São Paulo, São Paulo, Brazil; ^2^Departamento de Genética e Evolução, Universidade Federal de São Carlos, São Carlos, Brazil; ^3^Departamento de Parasitologia, Instituto de Ciências Biomédicas, Universidade de São Paulo, São Paulo, Brazil; ^4^Departamento de Ciência da Computação, Instituto de Matemática e Estatística, Universidade de São Paulo, São Paulo, Brazil; ^5^Departamento de Medicina Veterinária Preventiva e Saúde Animal, Faculdade de Medicina Veterinária e Zootecnia, Universidade de São Paulo, São Paulo, Brazil; ^6^Laboratory of Malaria and Vector Research, National Institute of Allergy and Infectious Diseases, Bethesda, MD, United States

**Keywords:** *Amblyomma*, tick, salivary glands, blood feeding, spotted fever, RNA-seq, transcriptome, proteome

## Abstract

Ticks are obligate blood feeding ectoparasites that transmit a wide variety of pathogenic microorganisms to their vertebrate hosts. *Amblyomma sculptum* is vector of *Rickettsia rickettsii*, the causative agent of Rocky Mountain spotted fever (RMSF), the most lethal rickettsiosis that affects humans. It is known that the transmission of pathogens by ticks is mainly associated with the physiology of the feeding process. Pathogens that are acquired with the blood meal must first colonize the tick gut and later the salivary glands (SG) in order to be transmitted during a subsequent blood feeding via saliva. Tick saliva contains a complex mixture of bioactive molecules with anticlotting, antiplatelet aggregation, vasodilatory, anti-inflammatory, and immunomodulatory properties to counteract both the hemostasis and defense mechanisms of the host. Besides facilitating tick feeding, the properties of saliva may also benefits survival and establishment of pathogens in the host. In the current study, we compared the sialotranscriptome of unfed *A. sculptum* ticks and those fed for 72 h on rabbits using next generation RNA sequencing (RNA-seq). The total of reads obtained were assembled in 9,560 coding sequences (CDSs) distributed in different functional classes. CDSs encoding secreted proteins, including lipocalins, mucins, protease inhibitors, glycine-rich proteins, metalloproteases, 8.9 kDa superfamily members, and immunity-related proteins were mostly upregulated by blood feeding. Selected CDSs were analyzed by real-time quantitative polymerase chain reaction preceded by reverse transcription (RT-qPCR), corroborating the transcriptional profile obtained by RNA-seq. Finally, high-performance liquid chromatography coupled with tandem mass spectrometry (LC-MS/MS) analysis revealed 124 proteins in saliva of ticks fed for 96–120 h. The corresponding CDSs of 59 of these proteins were upregulated in SG of fed ticks. To the best of our knowledge, this is the first report on the proteome of *A. sculptum* saliva. The functional characterization of the identified proteins might reveal potential targets to develop vaccines for tick control and/or blocking of *R. rickettsii* transmission as well as pharmacological bioproducts with antihemostatic, anti-inflammatory and antibacterial activities.

## Introduction

Ticks are obligate ectoparasites that infest numerous species of vertebrates. As result of their feeding on blood, these arthropods are versatile vectors of a wide variety of pathogenic microorganisms, such as viruses, bacteria, helminths, and protozoa (Jongejan and Uilenberg, 2004; Dantas-Torres et al., 2012; Otranto et al., 2013). Rocky Mountain spotted fever (RMSF), caused by *Rickettsia rickettsii*, is the most lethal tick-borne rickettsiosis that affects humans (Dantas-Torres, 2007). This disease is widely distributed in the Americas (Dantas-Torres, 2007; Walker, 2007; Labruna, 2009), with high case fatality rates (Angerami et al., 2006; Labruna, 2009). Different tick species have been implicated as *R. rickettsii* vectors, being *Dermacentor variabilis* and *Dermacentor andersoni* the main vectors in North America (Dantas-Torres, 2007). *Amblyomma americanum* and *Rhipicephalus sanguineus* are also implicated as vectors in the United States, respectively in the states of North Carolina and Arizona (Demma et al., 2005; Breitschwerdt et al., 2011). In Central and South America, the most important species that transmit *R. rickettsii* belong to the *Amblyomma cajennense* complex (Labruna, 2009; Nava et al., 2014). In the Brazilian territory, *Amblyomma sculptum* (formely named *A. cajennense*; Nava et al., 2014) and *Amblyomma aureolatum*, are incriminated as vectors (Labruna, 2009).

*Amblyomma sculptum* is widely distributed in Brazil, mainly in the southeast region. This tick species infests many species of both wild and domestic animals, although horses are the preferred hosts (Labruna et al., 2001). Capybaras (*Hydrochoerus hydrochaeris*) are also primary hosts of *A. sculptum*, mostly in RMSF endemic areas, being infested by all tick parasitic stages and acting as amplifier hosts (Labruna, 2009; Szabó et al., 2013). In addition, capybaras are susceptible to *R. rickettsii*, maintaining high bacteremia for several weeks and allowing infection of ticks (Souza et al., 2009). In the last decades, many ecological changes contributed to an increase in the populations of capybaras in the southeast region of Brazil, leading to an augment in *A. sculptum* density and, consequently, the re-emergence of RMSF (Labruna, 2009; Szabó et al., 2013). Importantly, besides transmission of rickettsiae, the bite of *A. sculptum* causes pain, severe inflammatory reaction, fever, and stress, resulting in significant economic losses (Oliveira et al., 2003).

The transmission of pathogens by ticks is mainly associated with the physiology of the feeding process and also with the vector immune system. Generally, the common route of pathogens acquired during the blood meal is the migration from the midgut (MG) to the haemocoel and, subsequently, the colonization of the salivary glands (SG) (Kazimírová and Štibrániová, 2013). Pathogens within the tick SG must then reach the saliva to be transmitted during a subsequent blood feeding. Tick saliva contains a complex mixture of bioactive molecules with anticlotting, antiplatelet aggregation, vasodilatory, anti-inflammatory, and immunomodulatory properties to counteract the host defense mechanisms (Hajdušek et al., 2013; Kazimírová and Štibrániová, 2013; Kotál et al., 2015; Šimo et al., 2017). Besides facilitating tick feeding, the antihemostatic and immunomodulatory properties of tick saliva may also benefit survival and establishment of pathogens in the host (Kazimírová and Štibrániová, 2013; Šimo et al., 2017). Therefore, the identification and characterization of bioactive molecules of tick SG and saliva might help to elucidate the molecular mechanisms of interaction between ticks, pathogens, and vertebrate hosts, revealing new vaccine targets to control ticks and the pathogens they transmit.

In the current study, the gene expression of the SG of unfed and 72 h fed *A. sculptum* was performed by next generation RNA sequencing (RNA-seq). The expression of selected coding sequences (CDSs) in SG of unfed, 24 and 72 h fed ticks was further analyzed by real-time quantitative polymerase chain reaction preceded by reverse transcription (RT-qPCR) in order to determine their temporal transcriptional profile. Finally, we determined the set of proteins contained in saliva of fed *A. sculptum* by high-performance liquid chromatography coupled with tandem mass spectrometry (LC-MS/MS). Data presented in this study amplify the knowledge of proteins possibly involved in tick feeding, which may also play a role on transmission of pathogens. Future functional studies to determine the role of such proteins on *A. sculptum* physiology as well as on the acquisition and transmission of *R. rickettsii* are warranted and might be useful to identify potential vaccine targets.

## Materials and methods

### Ethics statement

All procedures involving vertebrate animals were carried out according to the Brazilian National Law number 11794 and approved by the Institutional Animal Care and Use Committees from the Faculty of Veterinary Medicine and Zootecnics (protocol number 1423/2008) and the Institute of Biomedical Sciences (protocol number 128/2011), University of São Paulo, São Paulo, Brazil. Animal purchase and euthanasia procedures were performed as described in Galletti et al. (2013).

### Ticks and sample collection

Ticks were obtained from a laboratory colony of *A. sculptum* (Pedreira strain, São Paulo, Brazil). Larvae, nymphs, and adults were fed on rabbits (*Oryctolagus cuniculus*) as previously described (Pinter et al., 2002). Off-host phases were held in an incubator at 25°C and 95% of relative humidity. Adult females were manually removed from the vertebrate hosts after 24 or 72 h of feeding for dissection of SG. Firstly, ticks were washed in 70% ethanol and subsequently in sterile phosphate-buffered saline (PBS) (10 mM NaH_2_PO_4_, 1.8 mM KH_2_PO_4_, 140 mM NaCl, and 2.7 mM KCl, pH 7.4) for 10 min each. After dissection, SG were gently washed in sterile PBS and immediately transferred to 50 μL of RNA*later*® Stabilization Solution (Life Technologies, Carlsbad, CA, USA). The SG from unfed *A. sculptum* females (control) were dissected using the same procedure.

Salivation of females fed for 96–120 h on rabbits was induced by injection of approximately 1–3 μL of a solution of 50 mg/mL pilocarpine in 0.7 M NaCl into the tick hemocoel using a 12.7 × 0.33 mm needle BD Ultra-Fine™ (Becton Dickinson and Company, Franklin Lakes, NJ, USA) (Oliveira et al., 2013). The saliva was harvested every 10–15 min using a micropipette and transferred to a polypropylene tube kept in ice. Samples were stored at −80°C until use.

### RNA isolation, RNA-seq and bioinformatics analysis

The total RNA from tick SG was isolated using the NucleoSpin®TriPrep Kit (Macherey-Nagel, Düren, Germany) according to the manufacturer's specifications. The RNA extracted from 20 samples (each one composed by SG of three ticks) of each group (ticks unfed or fed for 72 h on rabbits) contributed equally for the composition of the pool RNA samples submitted to high throughput RNA-seq. Samples were tagged with specific barcodes and multiplex sequenced in four lanes using an Illumina HiSeq platform at the North Carolina State University facility (Raleigh, NC, USA).

Approximately 567 million reads of 101 base pairs were obtained using the single read mode [these reads also include the transcriptome of the MG of *A. sculptum* and *A. aureolatum* (Martins et al., 2017) and also of the SG of *A. aureolatum* data not shown]. Reads for each species were assembled using Abyss and Soapdenovo Trans programs with *K*-values varying from 21 to 91 (in 10 interval increments). Resulting assembly of reads were concatenated and clustered following the procedure described by Karim et al. (2011). CDSs were extracted based on matches to public databases or longer open reading frames with a signal peptide as an indicative of secretion (Karim et al., 2011). The comparison of the predicted amino acid sequences translated from the nucleotide sequences to the non-redundant protein database of the NCBI and to the Gene Ontology (GO) database (Ashburner et al., 2000) was performed using the blastx tool (Altschul et al., 1997). In addition, the tool reverse position-specific BLAST (rpsblast) (Altschul et al., 1997) was used to search for conserved protein domains in the Pfam (Bateman et al., 2000), SMART (Schultz et al., 2000), KOG (Tatusov et al., 2003), and conserved domains databases (CDD) (Marchler-Bauer et al., 2002). To identify putative secreted proteins, predicted protein products starting with a methionine residue were submitted to the SignalP server (Nielsen et al., 1997; Petersen et al., 2011). Glycosylation sites on the proteins were predicted by use of the program NetOGlyc (Julenius et al., 2005). The functional annotation of the CDSs was performed based on all the comparisons described above and their *e*-values. Finally, CDSs and their encoded proteins were classified based on function and/or protein families. Reads Per Kilobase Million (RPKM) values for deduced CDSs were calculated as described previously (Kotsyfakis et al., 2015b). Data were organized in a hyperlinked spreadsheet as described by Ribeiro et al. (2004). The complete table (Supplementary Table 1) with links may be downloaded from http://exon.niaid.nih.gov/transcriptome/Amb_sculptum/Supplementary_Table_1-Web.xlsx. The raw data were deposited to the Sequence Read Archives (SRA) of the National Center for Biotechnology Information (NCBI) under Bioproject number PRJNA343654. Only CDSs representing 90% of known proteins or larger than 250 amino acids were deposited to the Transcriptome Shotgun Annotation (TSA) portal of the NCBI [accession number GFAA00000000, version GFAA00000000.1; Biosamples SAMN05792022 (SG of unfed ticks) and SAMN05792023 (SG of ticks fed for 72 h)].

To compare the gene expression between unfed and fed ticks, paired comparisons of the number of reads hitting each contig were calculated by X^2^ tests to detect significant differences between samples when the minimum expected value was larger than 2 and *p* < 0.05. Normalized fold-ratios of the sample reads were computed by adjusting the numerator by a factor based on the ratio of the total number of reads in each sample, and adding one to the denominator to avoid division by zero.

The amino acid sequences of proteins encoded by selected CDSs (Acaj-56179, GenBank protein ID: JAU02549.1; Acaj-77950, Genbank protein ID: JAU02547.1; Acaj-65746, GenBank protein ID: JAU03230.1; and AcajSIGP-14784, Genbank protein ID: JAU02578.1) were used as query in blastp searches against Transcriptome Shotgun Assembly (tsa_nr; NCBI) database with the class Arachnida (taxid: 6854) as filter. The protein sequence of a given arachnid species with the best match with *A. sculptum* was selected as representative for that species (accession numbers available in Supplementary Table 2) and used to performing multiple sequence alignment (MSA) with the MUSCLE method (Edgar, 2004) and graphically edited using BioEdit software (Hall, 1999). Phylogenetic analysis of sequences was constructed with the Maximum Likelihood (ML) method with Jones-Taylor-Thornton (JTT) matrix-based substitution model (Jones et al., 1992) using MEGA 5 (Tamura et al., 2011) software. Node support of each clade was evaluated using a bootstrap analysis (1,000 replicates) (Felsenstein, 1985). The distances between the sequences (degree of similarity) are in the units of the number of amino acid substitutions per site (computed by the Poisson correction method) (Zuckerkandl and Pauling, 1965).

### RT-qPCR

Ten CDSs detected as differentially expressed by RNA-seq analysis were selected to be evaluated using RT-qPCR. One microgram of the total RNA extracted from the SG of unfed females or females fed for either 24 or 72 h on rabbits were treated with RQ1 RNase-free DNase (Promega, Madison, WI, USA). Resulting RNA was used as template for reverse transcription (RT) in complementary DNA (cDNA) using Platus transcriber RNase H-cDNA First Strand Kit (Sinapse–Inc, Miami, FL, USA) as described by the manufacturer.

Resulting cDNA was used as template in qPCR. The reactions were performed in a StepOne™ Plus System using SYBR® Green PCR Master Mix (equipment and reagent from Thermo Fisher Scientific, Waltham, MA, USA) and specific primers for selected CDSs (Supplementary Table 3) with cycling parameters of 95°C for 10 min followed by 40 cycles at 95°C for 15 s, 60°C for 60 s, and 72°C for 20 s. A melting curve analysis was carried out to check the specificity of the primers. Primers were designed using Primer3 (Rozen and Skaletsky, 2000). To determine the efficiency of each pair of primers, standard curves were generated using different concentrations of cDNA (400 to 3.12 ng; 2-fold dilution). Only primers presenting efficiency above 90% were used in the analyses. In addition, primer specificity was confirmed using DNA extracted from rabbit blood as control.

The 2^−ΔΔCt^ equation (Livak and Schmittgen, 2001) was utilized to calculate the relative expression (fold-change) of select CDSs in fed vs. unfed ticks. The CDS of the ribosomal protein S3A (Supplementary Table 3), constitutively expressed in SG of fed and unfed ticks (data not shown), was used as reference. Eight biological replicates (each one composed by RNA extracted from SG of three ticks) of each group (ticks fed for either 24 or 72 h and unfed ticks) were analyzed. All samples were analyzed in three technical replicates. The expression of a CDS was considered statistically different by comparing the median of the eight biological replicates of each group using the Wilcoxon test and *p*-values were corrected by the False Discovery Rate (FDR) method (Benjamini and Hochberg, 1995) for multiple tests. Difference in CDS expression was considered significant when *p* < 0.05. Spearman's correlation coefficient was calculated by GraphPad Prism software (GraphPad Software Inc., La Jolla, CA, USA) using log-transformed fold-change values from qPCR and RNA-seq experiments to verify the replicability between these two techniques.

### High-performance liquid chromatography coupled with tandem mass spectrometry (LC-MS/MS) and data analysis

Five samples containing the saliva of five ticks each (females fed for 96–120 h) were independently processed and analyzed. First, protein concentration in each sample was determined using a bicinchoninic acid protein assay (Pierce, Rockford, IL, USA). Proteins were submitted to extraction according to Mudenda et al. (2014), with modifications. After dilution in urea 8 M (1:10; v/v), proteins were reduced with DTT and alkylated with iodoacetamide. Urea was removed and the buffer was changed to 50 mM ammonium bicarbonate with 10% acetonitrile by ultrafiltration with a 10 kDa cutoff Amicon Ultra 0.5 mL filtration unit (Millipore). After digestion with trypsin, resulting peptides were submitted to LC-MS/MS analysis using an Orbitrap Fusion Tribrid (Thermo Fisher Scientific) mass spectrometer coupled with a Proxeon nano-LC through a nanoelectrospray ion source. Peptides were loaded onto an Acclaim™ PepMap™ 100 C_18_ pre-column (5 μm, 100 Å, 75 μm × 2 cm; Thermo Fisher Scientific) and separated on a PepMap™ RSLC C_18_ column (2 μm, 100 Å, 75 μm × 25 cm; Thermo Fisher Scientific) using a linear gradient of acetonitrile (0–40%) in 0.1% formic acid for 80 min at a flow rate of 300 nL/min. This gradient was followed by a 2 min increase to 80% acetonitrile and held at this concentration for 3 min. The nanoelectrospray voltage of the capillary was set to 1.8 kV. The analysis was performed in full scan (m/z 400–1,600) with the resolution of Orbitrap adjusted to 120,000. Using a 3 s cycle time and rapid scan, peptide ions with charge states 2–8 were fragmented in the linear ion trap using low-energy CID (normalized collision energy of 35%) and an isolation width of 1.6 dynamic exclusion was enabled with an exclusion duration of 20 s, and a repeat count of 1.

For protein identification, raw files were imported into PEAKS version 8.5 (Bioinformatics Solutions, Inc.,) and searched against *A. sculptum* transcript fasta database (9,560 CDSs presented in the current study) and the cRAP contaminant database (the common Repository of Adventitious Proteins—http://www.theGPM.org/crap), using a tolerance of 10 ppm for the precursor ion and 0.6 Da for fragment ions. Enzyme specificity was set to trypsin with a maximum of two missed cleavages and one non-specific cleavage. Carbamidomethylation of cysteine (57.021 Da) was set as a fixed modification, and oxidation of methionine (15.994 Da), and deamidation of asparagine and glutamine (0.98 Da) were selected as variable modifications. All peptide identifications were filtered in order to achieve a false discovery rate (FDR) of 0.5% using a decoy database approach. Contaminant proteins were filtered from the final list. The complete dataset was deposited to Pride database (accession number PXD007852).

## Results

### Gene expression profile of *A. sculptum* SG in response to blood feeding

The sialotranscriptomes of unfed and fed ticks were determined using high throughput RNA-seq. Resulting reads were assembled into 9,560 CDSs distributed in different functional classes (Table 1 and Supplementary Table 1). Over 29 million reads were obtained for unfed ticks, whereas 72 h fed ticks accounted almost 41 million reads (Table 1). Reads within transcription, protein synthesis, protein export, proteasome machineries, and transporters/storage functional classes were more represented in sialotranscriptome of unfed than of fed ticks (Table 1). On the other hand, reads of proteins predicted to be secreted corresponded to 36.53% of the sialotranscriptome of fed ticks (Table 1) and only to 10.95% of sialotranscriptome of unfed ticks (Table 1).

**Table 1:** Functional classification of CDSs of unfed and fed *A. sculptum* ticks.

	**SG-Unfed**			**SG-Fed (72 h)**		
**Functional class**	**Number of CDSs**	**Number of reads**	Relative abundance of reads^*^(%)	**Number of CDSs**	**Number of reads**	Relative abundance of reads^*^(%)
Cytoskeletal	301	1,108,899	3.79	304	907,896	2.23
Detoxification	85	58,478	0.20	92	126,600	0.31
Extracellular matrix/cell adhesion	291	2,025,509	6.93	302	3,084,348	7.58
Immunity	111	105,175	0.36	121	78,672	0.19
Amino acid metabolism	43	61,283	0.21	45	81,064	0.20
Carbohydrate metabolism	131	258,736	0.88	132	364,731	0.90
Energy metabolism	82	290,754	0.99	85	638,711	1.57
Intermediary metabolism	16	18,384	0.06	16	33,194	0.08
Lipid metabolism	183	258,154	0.88	196	305,605	0.75
Nucleotide metabolism	58	94,988	0.32	62	52,976	0.13
Nuclear export	32	310,812	1.06	32	119,450	0.29
Nuclear regulation	205	545,375	1.86	205	366,541	0.90
Protein export machinery	303	1,069,385	3.66	305	884,552	2.17
Protein modification machinery	192	434,173	1.48	197	1,061,107	2.61
Proteasome	176	819,051	2.80	176	505,338	1.24
Protein synthesis	287	8,053,986	27.54	288	4,133,884	10.16
Signal transduction	807	4,422,649	8.28	814	1,532,993	3.76
Storage	18	99,099	0.34	19	97,785	0.24
Transcription factor	131	406,243	1.39	132	222,886	0.55
Transcription machinery	425	2,686,898	9.19	426	1,426,038	3.50
Transporters and channels	381	1,226,355	4.19	397	793,366	1.95
Unknown	680	1,097,518	3.75	769	1,469,774	3.61
Unknown, conserved	1011	2,097,204	7.17	1024	3,759,621	9.24
Putative secreted	2314	3,202,168	10.95	2592	14,865,642	36.53
Transposable elements	544	314,820	1.08	567	961,273	2.36
Viral products	37	179,294	0.61	39	2,818,989	6.93
Total	8844	29,245,390		9337	40,693,036	

* *Relative abundance of reads: number of reads within each functional class in one specific sialotranscriptome (unfed ticks or ticks fed for 72 h) / total number of reads in the same sialotranscriptome*.

The majority of CDSs was shared between unfed and fed ticks (8,802 CDSs). Other CDSs were identified exclusively in sialotranscriptome of unfed or fed ticks. Among the 42 CDSs identified exclusively in the sialotranscriptome of unfed ticks, 18 are predicted to be secreted and all of them code unknown proteins. In addition, 535 CDSs are exclusive of the sialotranscriptome of fed ticks, among which 296 encode putative secreted proteins. Two thirds of the CDSs of putative secreted proteins detected only in sialotranscriptome of fed ticks present unknown function and the remaining belongs to well-known protein families, such as protease inhibitors, metalloproteases, and lipocalins (Supplementary Table 1).

**Figure 1:**
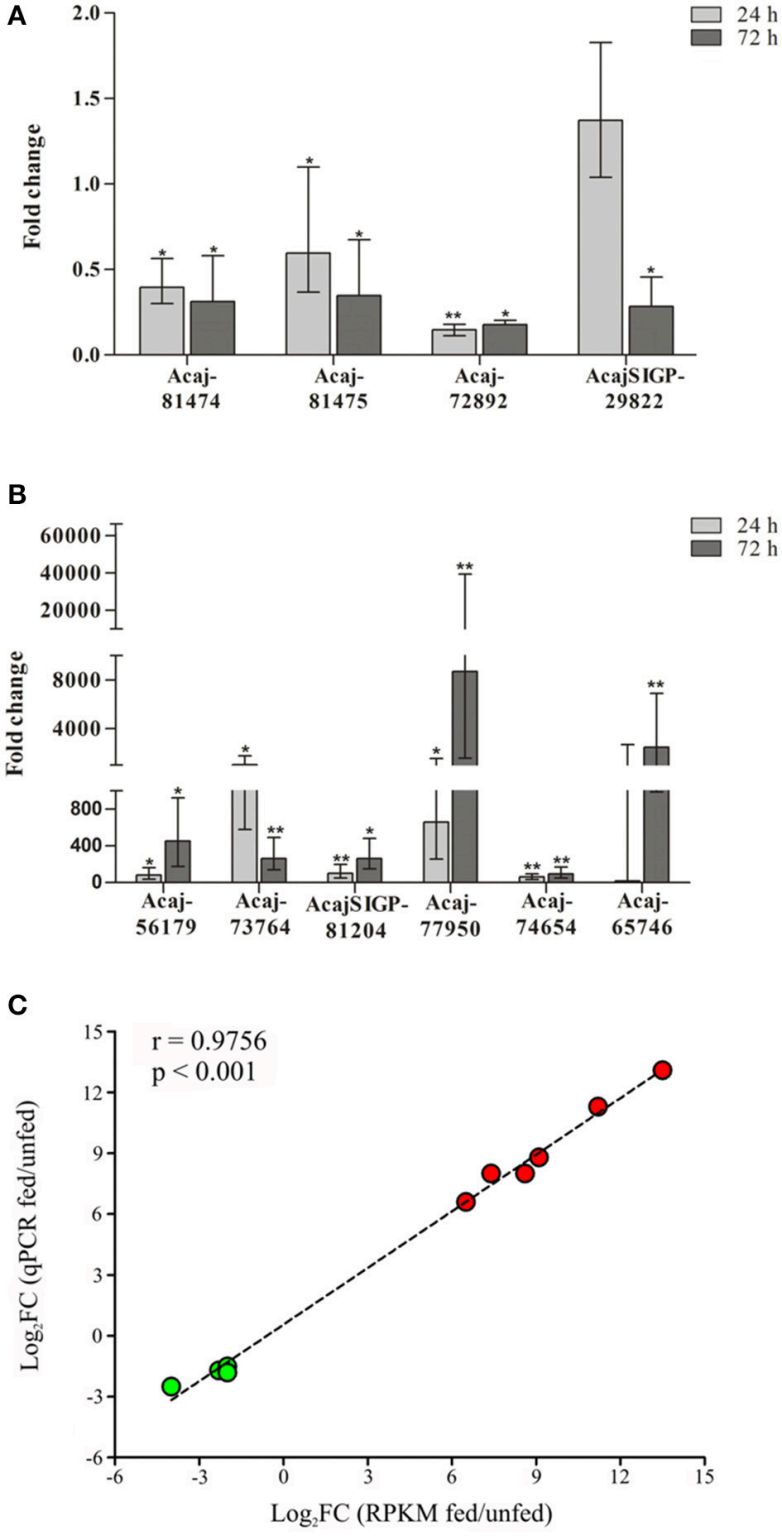
Temporal transcriptional analysis of selected CDSs in SG of unfed and fed ticks by RT-qPCR. **(A)** Downregulated and **(B)** upregulated CDSs identified by RNA-seq were selected for RT-qPCR analysis. The expression levels of CDSs in ticks fed for either 24 or 72 h was compared to expression in SG of unfed ticks (control) using the 2^−ΔΔCt^ method (Livak and Schmittgen, 2001). Error bars represent 95% of confidence interval; ^*^p < 0.05 and ^**^p < 0.001 are corrected for multiple comparisons by the False Discovery Rate (FDR). **(C)** Spearman correlation between the expression data determined by RT-qPCR and RNA-seq (RPKM values). The fold-changes obtained by either qPCR (y-axis) or RNA-seq (x-axis) was plotted with log-transformed values; therefore negative values means downregulation (CDSs represented by green symbols), while positive values means upregulation (CDSs represented by red symbols). The dashed line represents the goodness of fit of the data calculated by linear regression analysis.

The expression of 10 CDSs identified in the sialotranscriptome as differentially expressed by tick feeding was further assessed by RT-qPCR (Figure 1 and Table 2). The downregulation of sequences encoding glycine-rich proteins (Acaj-81474 and Acaj-81475), eukaryotic translation initiation factor 4 gamma (Acaj-72892), and tick cystatin 1 (AcajSIGP-29822) was confirmed by RT-qPCR (Figure 1A and Table 2). The transcript levels of all of these CDSs were already significantly lower in SG of ticks fed for 24 h (Figure 1A and Table 2), except for AcajSIGP-29822, which was not differentially expressed in ticks fed for 24 h, with significant differences in relation to the control (unfed ticks) only in ticks fed for 72 h (Figure 1A and Table 2). The upregulation of sequences encoding putative secreted cysteine rich protein containing trypsin inhibitor-like (TIL) domain (Acaj-56179), glycine-rich proteins (Acaj-73764 and Acaj-74654), peptidoglycan recognition protein (PGRP) (AcajSIGP-81204), tick Kunitz inhibitor (Acaj-77950), and putative secreted SG antimicrobial peptide (AMP) similar to defensin (Acaj-65746) was also confirmed by RT-qPCR (Figure 1B and Table 2). The transcript levels of all of these CDSs were higher in SG of ticks fed for either 24 or 72 h than in unfed ticks, although the upregulation of the defensin (Acaj-65746) was significant only after 72 h (Figure 1B and Table 2). The correlation analysis showed that the gene expression regulation upon blood feeding was highly positively correlated between RNA-seq and RT-qPCR measurements (*r* = 0.9756, *p* < 0.0001), strengthening the transcriptional findings of the current study (Figure 1C).

**Table 2:** Relative expression of selected CDSs in SG of fed ticks in relation to unfed ticks by RNA-seq and RT-qPCR.

**CDSs**	**Annotation**	**RNA-seq**	**RT-qPCR**	
		**72 h**	**72 h**	**24 h**
Acaj-81474	Glycine-rich cell wall structural protein	0.20^*^	0.31^*^	0.40^*^
Acaj-81475	Glycine-rich cell wall structural protein	0.24^*^	0.35^*^	0.60^*^
Acaj-72892	Eukaryotic translation initiation factor 4 gamma	0.06^*^	0.18^*^	0.15^**^
AcajSIGP-29822	Tick_cistatins_1	0.25^*^	0.28^*^	1.37
Acaj-56179	Secreted cysteine rich protein partial	*3, 443*.20^*^	454.77^*^	83.06^*^
Acaj-73764	Glycine-rich cell wall structural protein 2	164.99^*^	262.06^**^	*1, 032*.91^*^
AcajSIGP-81204	Peptidoglycan recognition protein	374.91^*^	263.88^*^	100.78^**^
Acaj-77950	Tick_Kunitz_135	*7, 868*.61^*^	*8, 694*.17^**^	660.38^*^
Acaj-74654	Glycine-rich cell wall structural protein	89.75^*^	95.75^**^	62.79^**^
Acaj-65746	Secreted salivary gland peptide	*1, 177*.56^*^	*2, 460*.95^**^	16.51

* *p < 0.05;*

** *p < 0.001 [Statistically significant differences of expression in the SG of fed ticks in relation to the control (unfed ticks)]*.

### Transcription of putative secreted proteins in SG of *A. sculptum* ticks during feeding and secretion to saliva

**Figure 2:**
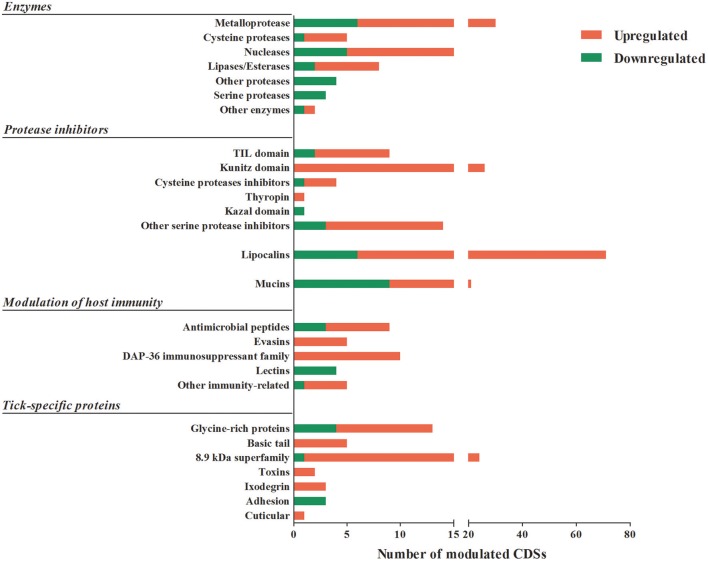
Putative secreted proteins CDSs differentially expressed by feeding in *A. sculptum* SG. The total number of CDSs of proteins predicted to be secreted that were significantly down (green) or upregulated (red) by blood feeding in SG of *A. sculptum* are presented.

**Figure 3:**
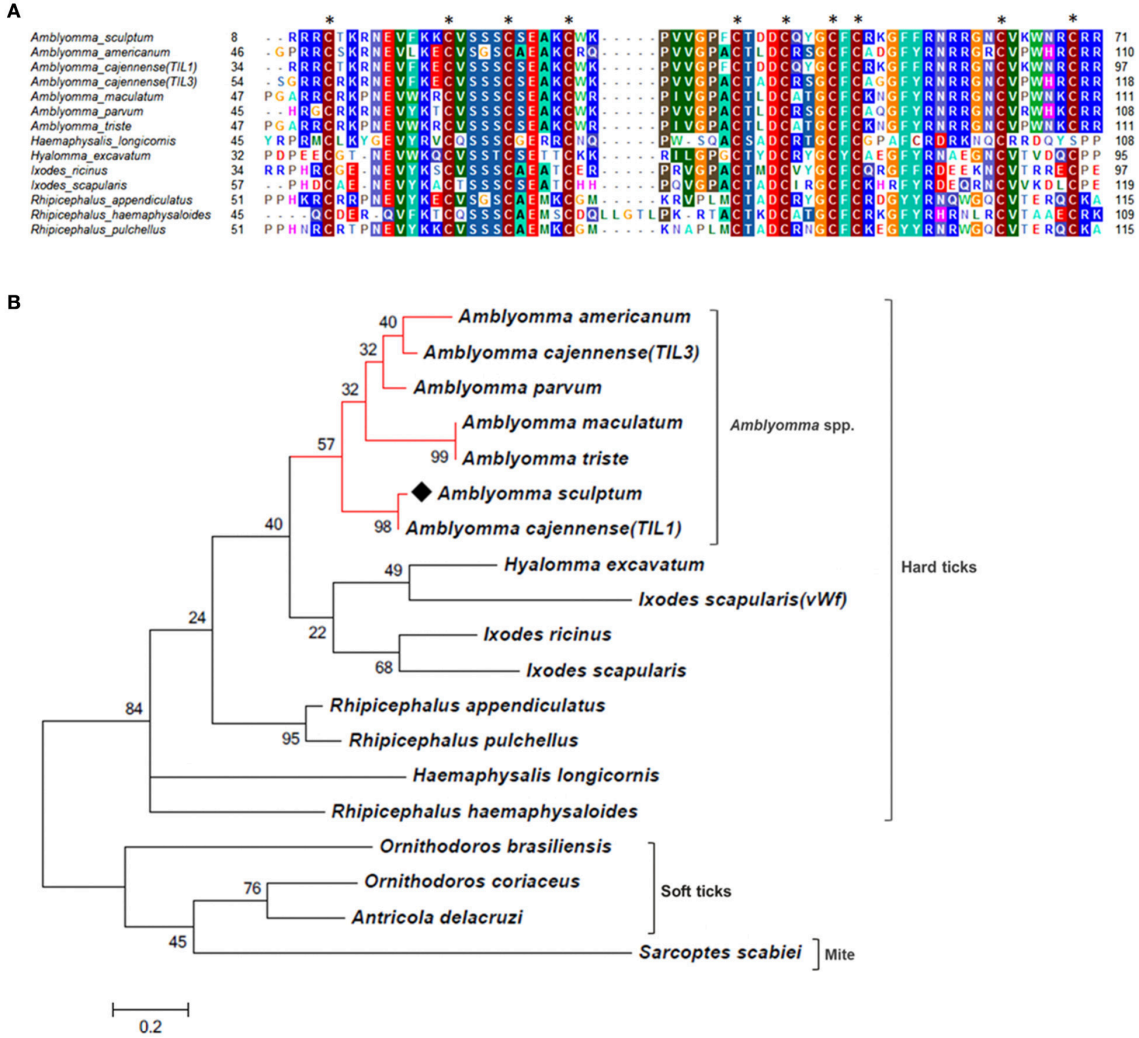
MSA and phylogenetic analysis of TIL domain containing proteins. **(A)** Multiple sequence alignment of protein sequences was performed using MUSCLE method. The numbers flanking the alignment represent the start (left) and end (right) amino acid position of each sequence in the protein domain. Asterisks highlight the conserved cysteine residues. Threshold for shading colors of amino acid similarity was 40%. **(B)** A phylogenetic tree was constructed with protein sequences from ticks and mite using Maximum Likelihood (ML) method. Numbers next to the branches represent the percentage of replicate trees in which the associated taxa clustered together in the bootstrap test (1,000 replicates). The tree is drawn to scale, with branch lengths measured in the number of substitutions per site. The analysis involved 19 amino acid sequences (accession numbers available in Supplementary Table 2). All positions containing gaps and missing data were eliminated. There were a total of 51 positions in the final dataset. Bar scale at the bottom indicates 20% amino acid divergence. Diamond symbol refers to the CDS Acaj-56179 from the sialotranscriptome of *A. scultpum* identified in this work.

**Figure 4:**
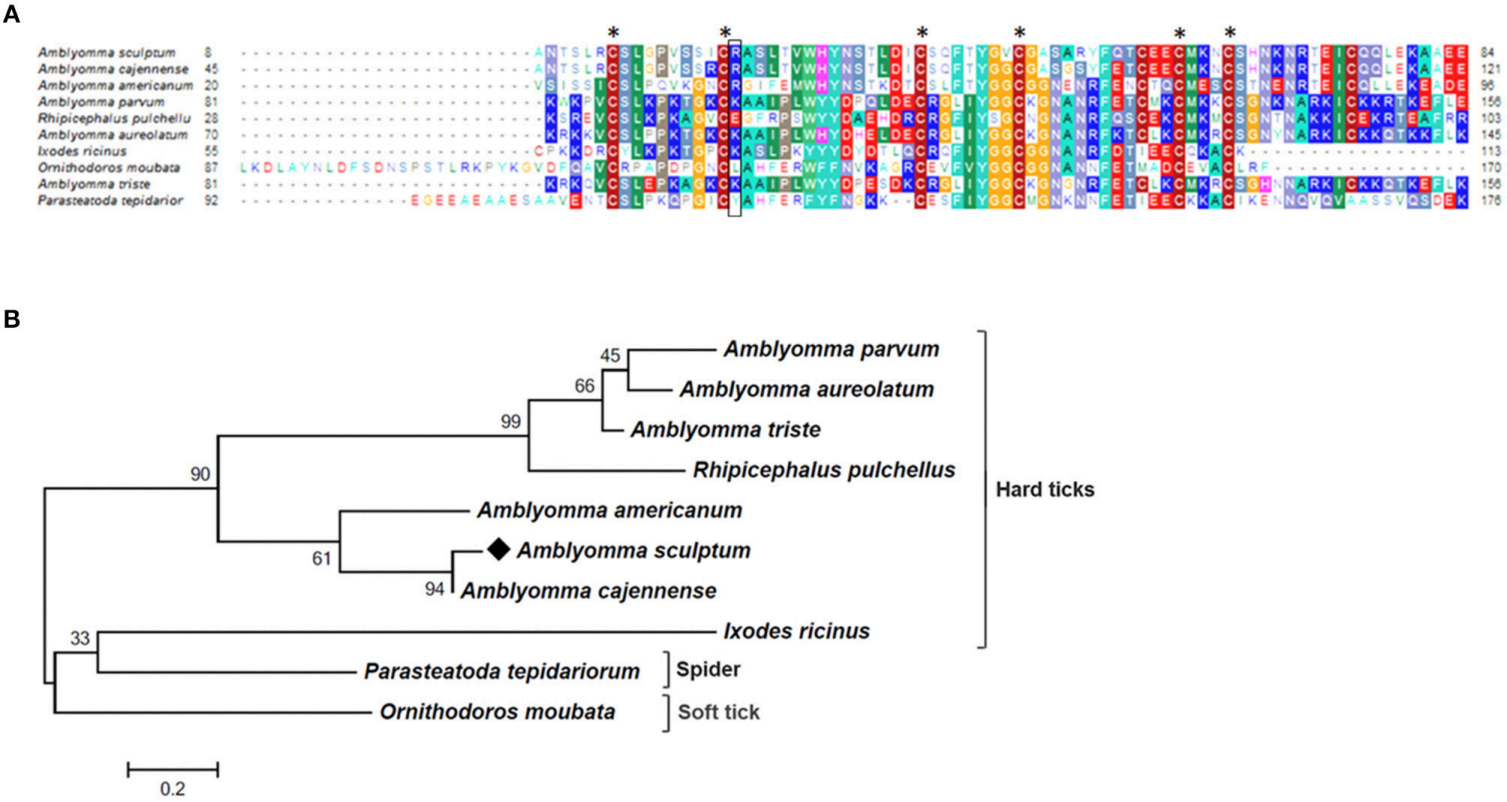
MSA and phylogenetic analysis of Kunitz domain containing proteins. **(A)** Multiple sequence alignment of protein sequences was performed using MUSCLE method. The numbers flanking the alignment represent the start (left) and end (right) amino acid position of each sequence in the protein domain. Asterisks highlight the conserved cysteine residues, while black box correspond to P1 site. Threshold for shading colors of amino acid similarity was 40%. **(B)** A phylogenetic tree was constructed with protein sequences from ticks and spider using Maximum Likelihood (ML) method. Numbers next to the branches represent the percentage of replicate trees in which the associated taxa clustered together in the bootstrap test (1,000 replicates). The tree is drawn to scale, with branch lengths measured in the number of substitutions per site. The analysis involved 10 amino acid sequences (accession numbers available in Supplementary Table 2). All positions containing gaps and missing data were eliminated. There were a total of 64 positions in the final dataset. Bar scale at the bottom indicates 20% amino acid divergence. Diamond symbol refers to the Acaj-77950 from the sialotranscriptome of the *A. scultpum* identified in this work.

As mentioned above, the majority of sequences encoding proteins predicted to be secreted were upregulated by blood feeding (Table 3, Figure 2 and Supplementary Table 1). Among them, we highlight members of lipocalin, mucin, metalloprotease, 8.9 kDa, and serine protease inhibitors families as well as immunity-related proteins (such as AMPs) (Table 3). Among 72 lipocalin CDSs found in the *A. sculptum* sialotranscriptome, 65 were upregulated by blood feeding (Figure 2). Similarly, 12 out of 36 mucin CDSs were upregulated (Figure 2). In addition, 24 CDSs of metalloproteases were upregulated and only six were downregulated (Figure 2). The sialotranscriptome of *A. sculptum* also revealed the upregulation of several protease inhibitor transcripts by blood feeding, the majority belonging to TIL and Kunitz families. Among the 39 CDSs representing those protease inhibitors, 33 were upregulated by feeding (Figure 2). Components of both inhibitor families were previously reported to exhibit antimicrobial properties in ticks (Fogaça et al., 2006; Ceraul et al., 2008). The annotation for the protein encoded by the CDS Acaj-56179 in protein domains databases (Pfam ID 01826 and UniprotKB/Swiss-Prot ID P83516, Supplementary Table 1) shows that it possesses the key features of TIL domain containing proteins. TIL domain is typically composed of five disulfide bonds formed by 10 cysteine residues in a stretch of approximately 54 amino acid residues (Bania et al., 1999), necessary for their biological properties, which include both antimicrobial and serine protease inhibitory activities (Fogaça et al., 2006; Wang et al., 2015). The MSA analysis of the amino acid sequence deduced from the CDS Acaj-56179 with similar sequences of other arachnids (Figure 3A) illustrated the highly conserved feature of the 10 cysteine residue positions among the sequences of hard ticks (family Ixodidae). The relationships of these sequences with sequences of soft ticks (family Argasidae) and the mite *Sarcoptes scabiei* showed the expected main clades composed by species of the families Ixodidae and Argasidae separately (Figure 3B), and the mite sequence placed into the soft tick branch as outer group. Importantly, TIL domain containing proteins from *Amblyomma* species were similar enough to constitute a unified subclade (red branch, Figure 3B). The annotation for the protein encoded by the CDS Acaj-77950 in protein domains databases (Pfam ID 00014 and UniprotKB/Swiss-Prot ID Q9WU03, Supplementary Table 1) showed that it is member of the Bovine pancreatic trypsin inhibitor (BPTI) family. MSA analysis showed the conserved disposition of the six cysteine residues of Kunitz domain (Ranasinghe and McManus, 2013) of Acaj-77950 and all similar sequences of other arachnids (Figure 4A). Regarding sequence relationships, the analysis showed that *Ixodes ricinus* (hard tick) is closer to the spider *Parasteatoda tepidariorum* and to the soft tick *Ornithodorus moubata* than to the other analyzed hard ticks (Figure 4B), which reflect the dissimilarity of *I. ricinus* and *O. moubata* sequences in relation to the other tick sequences observed in MSA analysis (Figure 4A).

**Table 3:** Functional classification of CDSs of putative secreted proteins in unfed and fed *A. sculptum* ticks.

**Families of putative secreted proteins**	**Number of CDSs per protein family**	**Total reads in SG unfed**	**Relative abundance of reads (%)**	**Total reads in SG Fed (72 h)**	**Relative abundance of reads (%)**
**ENZYMES**					
Metalloproteases	33	130,620	4.08	953,503	6.41
Cysteine proteases	7	22,069	0.69	198,909	1.34
Nucleases	22	21,884	0.68	133,781	0.90
Lipases/Esterases	17	14,441	0.45	24,844	0.17
Other proteases	9	16,156	0.50	21,342	0.14
Serine proteases	6	23,930	0.75	15,524	0.10
Other enzymes	2	2,351	0.07	4,701	0.03
**PROTEASE INHIBITORS**					
TIL domain	10	9,733	0.30	182,071	1.22
Kunitz domain	29	11,061	0.35	129,613	0.87
Cysteine protease inhibitors	6	2,577	0.08	11,862	0.08
Thyropin	2	868	0.03	3,576	0.02
Kazal domain	2	589	0.02	1,190	0.01
Other serine protease inhibitors	16	74,175	2.32	1,587,200	10.68
Lipocalins	72	8,456	2.64	570,185	3.84
Mucins	36	111,191	3.47	244,246	1.64
**IMMUNITY**					
Antimicrobial peptides	12	2,462	0.08	43,771	0.29
Evasins	7	2,844	0.09	30,023	0.20
Da-p36 immunosuppressant family	10	130	0.00	29,798	0.20
Lectins	8	16,069	0.50	15,142	0.10
Other immunity-related	6	13,332	0.42	77,167	0.52
**TICK-SPECIFIC PROTEINS**					
Glycine-rich proteins	17	32,252	10.07	3,325,247	22.37
Basic tail	6	72,556	2.27	469,358	3.16
8.9 kDa superfamily	24	4,095	0.13	118,235	0.80
Toxins	3	968	0.03	47,120	0.32
Ixodegrin	5	2,509	0.08	36,674	0.25
Adhesion	3	2,520	0.08	1,481	0.01
Cuticular	1	59	0.00	556	0.00
Novel putative secreted	82	116,574	3.64	132,096	0.89
Other putative secreted	1060	545,944	17.05	4,029,020	27.10
Unknown putative conserved secreted	103	143,033	4.47	364,287	2.45
Unknown putative secreted	1084	1,430,348	44.67	2,063,120	13.88
**Total Secreted**		3,202,168		14,865,642	

The 8.9 kDa superfamily is composed of proteins exclusively found in hard ticks, but none of its members were functionally characterized so far (Francischetti et al., 2009; Karim et al., 2011). Importantly, 23 CDSs of members of this family were upregulated by blood feeding in *A. sculptum* SG (Table 3 and Figure 2). Glycine-rich proteins correspond to another family of proteins that are specifically found in ticks. Seventeen CDSs of glycine-rich proteins were identified in *A. sculptum* sialotranscriptome (Table 3), among which nine were upregulated by feeding (Figure 2).

**Figure 5:**
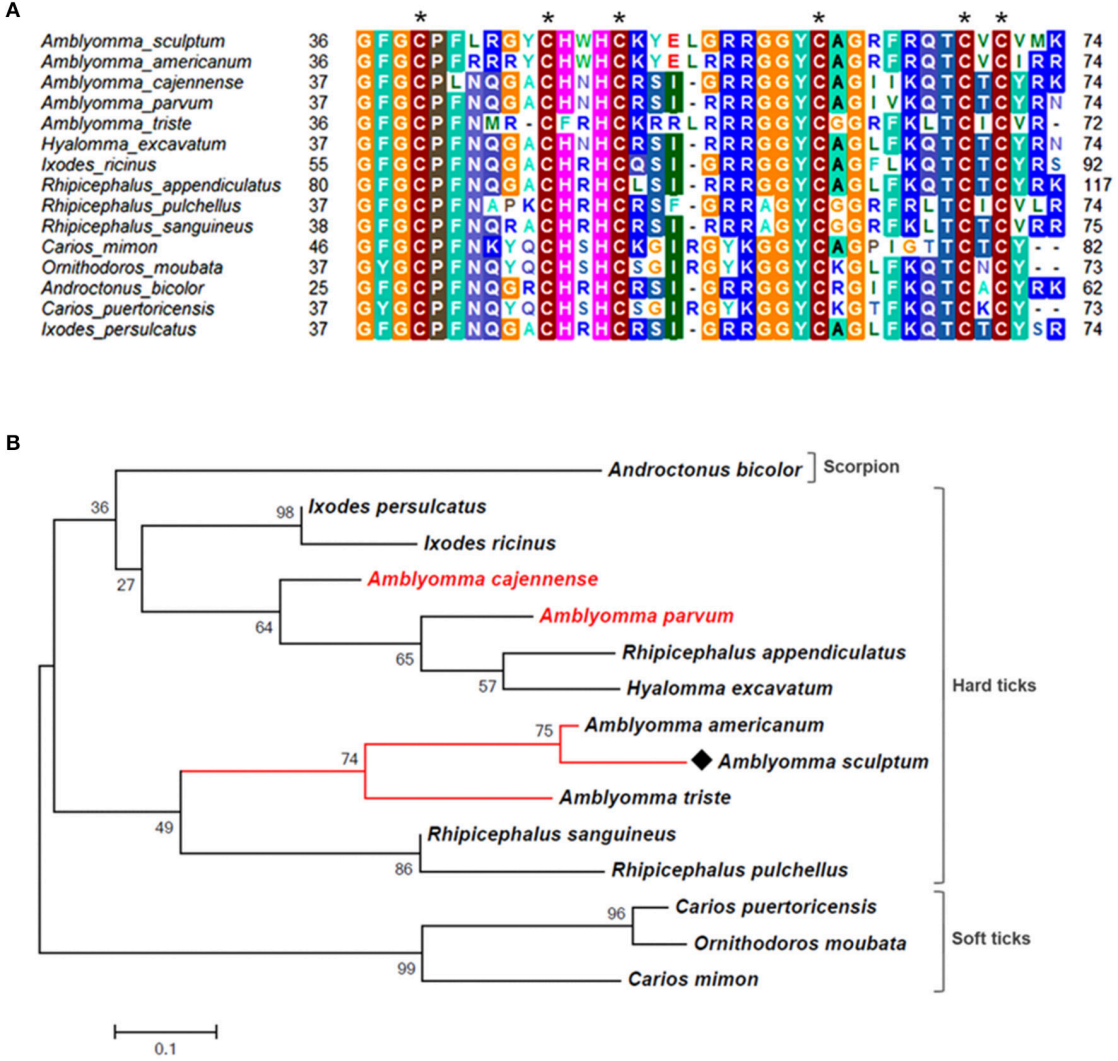
MSA and phylogenetic analysis of defensins. **(A)** Multiple sequence alignment of protein sequences was performed using MUSCLE method. The numbers flanking the alignment represent the start (left) and end (right) amino acid position of each sequence in the protein domain. Asterisks highlight the conserved cysteine residues. Threshold for shading colors of amino acid similarity was 50%. **(B)** A phylogenetic tree was constructed with protein sequences from ticks and scorpion using Maximum Likelihood (ML) method. Numbers next to the branches represent the percentage of replicate trees in which the associated taxa clustered together in the bootstrap test (1,000 replicates). The tree is drawn to scale, with branch lengths measured in the number of substitutions per site. The analysis involved 15 amino acid sequences (accession numbers available in Supplementary Table 2). All positions containing gaps and missing data were eliminated. There were a total of 58 positions in the final dataset. Bar scale at the bottom indicates 10% amino acid divergence. Diamond symbol refers to the CDS Acaj-65746 from the sialotranscriptome of *A. scultpum* identified in this work.

The majority of sequences encoding tick immune system components was also upregulated by feeding in SG of *A. sculptum*, except for lectins (Figure 2). The CDSs of one defensin (Acaj-65746) was highly upregulated in SG of fed ticks (Supplementary Table 1, Table 2, and Figure 1B). The annotated information for this sequence in protein domains databases (Pfam ID 01097 and UniprotKB/Swiss-Prot ID Q86QI5, Supplementary Table 1) shows that it is member of the arthropod defensin family, also named as Knottin scorpion toxin-like in InterPro database (Gracy et al., 2008). The mature peptide chain of member of this family ranges from 38 to 51 amino acids in length with six conserved cysteine residues involved in three-disulfide bonds. The conserved cysteine residues can be observed through the alignment of Acaj-65746 with related proteins (Figure 5A). The phylogenetic tree resembled the Ixodidae and Argasidae taxonomic clades (Figure 5B). Interestingly, the defensin of *Androctonus bicolor* (the only arachnid sequence besides ticks that retrieved from blast analysis) was grouped into hard ticks main clade and not as an outer group, showing that defensins of hard ticks are more similar to scorpion than to soft ticks.

Among the sequences with putative host immunomodulatory activity, 10 CDSs of the Da-p36 immunosuppressant family were identified (Table 3) and all of them were upregulated by feeding (Figure 2). Five evasin CDSs were also detected as upregulated by feeding in *A. sculptum* SG (Table 3 and Figure 2).

As expected, we also observed a high number of CDSs (2,329) encoding putative secreted proteins classified as (i) novel putative secreted (CDSs of unknown products), (ii) other putative secreted (CDSs of other classes of annotated putative secreted proteins), (iii) unknown putative conserved (CDSs of conserved putative secreted protein precursors) and (iv) unknown putative secreted proteins (CDSs of hypothetical putative secreted protein precursors; Table 3).

**Figure 6:**
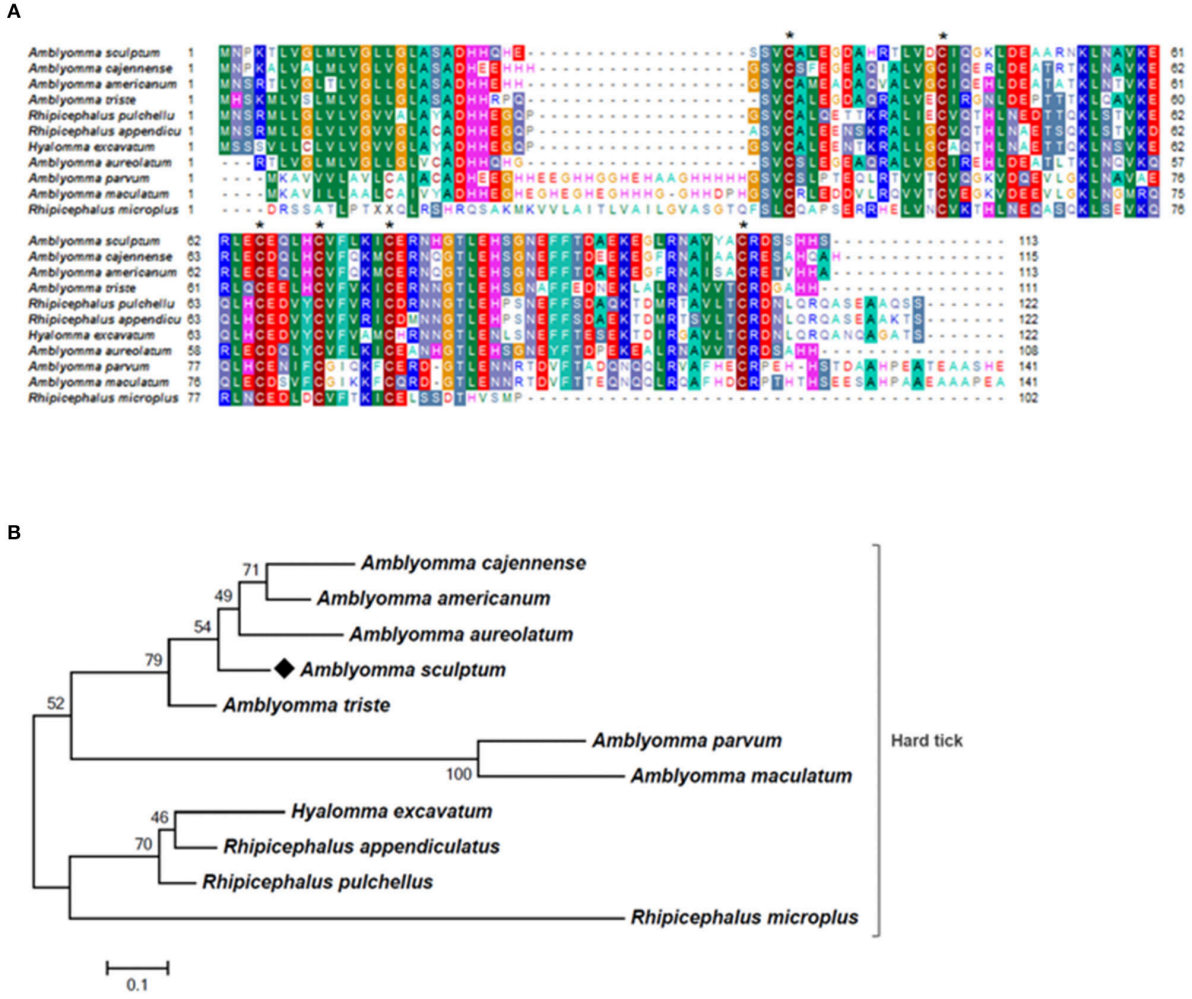
MSA and phylogenetic analysis of microplusin-like AMPs. **(A)** Multiple sequence alignment of protein sequences using MUSCLE method. The numbers flanking the alignment represent the start (left) and end (right) amino acid position of each sequence in the protein domain. Asterisks highlight the conserved cysteine residues. Threshold for shading colors of amino acid similarity was 40%. **(B)** A phylogenetic tree was constructed with protein sequences from ticks using Maximum Likelihood (ML) method. Numbers next to the branches represent the percentage of replicate trees in which the associated taxa clustered together in the bootstrap test (1,000 replicates). The tree is drawn to scale, with branch lengths measured in the number of substitutions per site. The analysis involved 11 amino acid sequences (accession numbers available in Supplementary Table 2). All positions containing gaps and missing data were eliminated. There were a total of 75 positions in the final dataset. Bar scale at the bottom indicates 10% amino acid divergence. Diamond symbol refers to the AcajSIGP-14784 from the sialotranscriptome of *A. scultpum* identified in this work.

To identify the proteins encoded by *A. sculptum* SG that are secreted into the saliva, the proteome of the saliva of fed ticks was determined by LC-MS/MS. One hundred twenty-four proteins were identified (Table 4 and Supplementary Table 4), whose all transcripts were detected in sialotranscriptome. Importantly, 58 of these proteins belong to the putative secreted protein functional class (Table 4). Twenty-three of these putative secreted proteins correspond to proteins with non-annotated function (Supplementary Table 4). Regarding putative secreted proteins with annotated function, we highlight six lipocalins, four 8.9 kDa proteins, three glycine-rich proteins, and three AMPs. The corresponding CDSs of five from six lipocalins detected in tick saliva were upregulated in SG of fed ticks (Supplementary Tables 1, 4). Regarding 8.9 kDa proteins, all corresponding CDSs presented high transcriptional levels in SG of fed ticks, while only one CDS of the three glycine-rich proteins detected in tick saliva was upregulated (Supplementary Tables 1, 4). Three histidine-rich AMPs similar to microplusins (Fogaça et al., 2004; Lai et al., 2004) were also detected in tick saliva (Supplementary Table 4). The corresponding CDSs of two of these proteins were upregulated in SG of fed ticks (ACAJ-77500 and ACAJSIGP-14784), while the third CDS (Acaj-57400) was not modulated (Supplementary Tables 1, 4). The protein encoded by the CDS AcajSIGP-14784 was also detected in saliva of ticks fed for 8 days on rabbits (data not shown). The MSA analysis of this peptide with similar sequences of other ticks showed that they share the six conserved cysteine residues (Figure 6A), a characteristic feature of microplusins (Fogaça et al., 2004; Lai et al., 2004). Sequence relationships showed that sequences of all *Amblyomma* species were grouped in one clade, while sequences of other species were grouped in a distinct clade (Figure 6B). The most divergent sequence in the later clade is the microplusin identified in the saliva of *Rhipicephalus microplus* (Tirloni et al., 2014; Figure 6B).

**Table 4:** Functional classification of proteins detected in saliva of fed *A. sculptum* ticks.

**Functional class**	**Number of proteins**
Cytoskeletal	18
Extracellular matrix/cell adhesion	08
Immunity	02
Carbohydrate metabolism	02
Energy metabolism	01
Intermediary metabolism	01
Lipid metabolism	01
Nuclear regulation	02
Protein export machinery	01
Protein modification machinery	14
Protein synthesis	02
Signal transduction	05
Storage	08
Putative secreted	58
Viral products	01
Total	124

## Discussion

During the tick feeding, SG of hard ticks are able to concentrate blood nutrients by returning the excess of water and also ions to the host via saliva (Bowman and Sauer, 2004; Šimo et al., 2017). The tick saliva also contains a cocktail of antihemostatic, anti-inflammatory and immunomodulatory molecules, guaranteeing the blood meal acquisition (Francischetti et al., 2009; Hajdušek et al., 2013; Kazimírová and Štibrániová, 2013; Kotál et al., 2015; Chmelar et al., 2016b; Šimo et al., 2017). Due to the importance of SG to tick feeding, we compared the sialotranscriptomes of unfed and fed *A. sculptum* ticks. Transcripts of most of the identified CDSs were detected in both sialotranscriptomes, although certain CDSs were found exclusively in only one sialotranscriptome. The sialotranscriptome of fed ticks presented the majority of these exclusive CDSs, suggesting that the proteins encoded by these sequences might play an important role during the feeding process.

In general, transcripts within protein synthesis and transcription machinery classes showed a higher proportion in SG of unfed *A. sculptum* than in fed ticks, suggesting a downregulation effect of blood feeding on protein expression. On the other hand, sequences coding proteins predicted to be secreted were mostly upregulated by blood acquisition. In accordance to our data, putative secreted protein transcripts were also more abundant after feeding in SG of female *Amblyomma maculatum* (Karim et al., 2011) and *A. americanum* (Karim and Ribeiro, 2015). Transcription of selected CDSs were analyzed by RT-qPCR. The high correlation between RNA-seq and qPCR data strengthens the transcriptional findings of the present study.

It is well-known that tick feeding triggers host defense mechanisms, such as hemostasis, inflammation, and immune responses. The SG of ticks, in turn, secrete several molecules into saliva to counteract, modulate and evade host immune responses, ensuring a successful feeding (Chmelar et al., 2012, 2016a,b; Kotál et al., 2015; Šimo et al., 2017). Indeed, sequences encoding putative secreted proteins that present antihemostatic, anti-inflammatory, and immunomodulatory properties, such as members of lipocalin, metalloprotease, and protease inhibitor families were significantly upregulated in SG of *A. sculptum* by feeding.

Lipocalins are anti-inflammatory proteins that bind both histamine and serotonin (Paesen et al., 1999; Sangamnatdej et al., 2002; Francischetti et al., 2009). It has been previously shown that elevated concentrations of histamine on the feeding site can affect tick attachment, feeding efficiency, and reproductive success, as demonstrated for *D. andersoni* (Paine et al., 1983) and *R. microplus* (Kemp and Bourne, 1980). Accordingly, treatment of infested animals with histamine antagonists was shown to improve tick engorgement and reduce acquired resistance to tick feeding (Tatchell and Bennett, 1969; Wikel, 1982). Therefore, the upregulation of lipocalins seems to be important to prevent excessive plasma exudation, inflammation and grooming behavior associated to vasoactive amines, thus allowing ticks to efficiently acquire the blood meal.

Transcription of metalloproteases was also significantly upregulated in sialotranscriptome of *A. sculptum* by feeding. Some previous studies have reported the expression of metalloproteases in tick SG (Valenzuela et al., 2002; Harnnoi et al., 2007; Decrem et al., 2008). The metalloproteases found in tick sialotranscriptomes belong to the reprolysin family (Francischetti et al., 2005b; Harnnoi et al., 2007; Mans et al., 2008), which present high similarity to the hemorrhagic snake venom metalloproteases (SVMPs) (Francischetti et al., 2003, 2005a). Therefore, it is possible that these proteins promote the fluidity of the blood in the feeding site during the long-extended feeding, by performing antihemostatic activities, such as fibrinogenolysis and fibrinolysis (Francischetti et al., 2003; Barnard et al., 2012). Importantly, the immunization of bovines with the reprolysin BrRm-MP4 of *R. microplus* decreased both feeding and reproductive rates of females (Ali et al., 2015), highlighting the potential of metalloproteinases as vaccine candidates.

The analysis of the sialotranscriptome of *A. sculptum* also revealed the presence of several protease inhibitor transcripts, the majority belonging to TIL and Kunitz families. Protease inhibitors has also been extensively described in SG of ticks (Francischetti et al., 2009; Chmelar et al., 2017). It has been previously shown that these molecules play an important role during tick feeding, preventing host blood clotting and ensuring acquisition of a blood meal (Francischetti et al., 2002, 2004, 2005a; Sasaki et al., 2004; Cao et al., 2013). It is known that ixodidin, a TIL domain containing protein isolated from the hemocytes of *R. microplus*, presents antimicrobial properties besides inhibiting the activity of serine proteases (Fogaça et al., 2006). In addition, it was previously reported that one Kunitz inhibitor of *D. variabilis* exhibits bacteriostatic effect against *Rickettsia montanensis* (Ceraul et al., 2008) and that its knockdown by RNA interference (RNAi) increases the tick susceptibility to infection (Ceraul et al., 2011). Interestingly, MSA and phylogenetic analyses of both TIL and Kunitz domain containing proteins of *A. sculptum* (Figures 3, 4, respectively) showed that they possess the key features required for biological properties of such molecules, suggesting that they may also exhibit antimicrobial and serine proteinase inhibitory activities.

The mucin family is the second class of putative secreted proteins mostly represented in *A. sculptum* sialotranscriptome. The members of this family are serine-and/or threonine-rich secreted proteins that have an O-N-acetylgalactosylation site in common (Karim et al., 2011). Because of dense glycosylation and hydration capacity, mucins can act as protective barriers, providing lubrication of various tick tissues (Hang and Bertozzi, 2005). Therefore, it is plausible to suppose that mucins may play important role in blood acquisition, maintaining the integrity of tick mouthparts (Ribeiro et al., 2006; Anderson et al., 2008; Anatriello et al., 2010).

The transcriptomes of *A. sculptum* evidenced that distinct members of the same protein family present a higher transcriptional level in SG of either fed or unfed ticks. For instance, 65 out of 72 CDSs of lipocalins were upregulated and seven were downregulated by blood feeding. A similar pattern was observed for other protein families, such as mucins, metalloproteinases, and protease inhibitors. In fact, recent studies that evaluated time-dependent expression of proteins by tick SG have found similar results for *I. ricinus* (Kotsyfakis et al., 2015b), *Ixodes scapularis* (Kim et al., 2016), and *A. americanum* (Karim and Ribeiro, 2015; Bullard et al., 2016). Therefore, it is possible that *A. sculptum*, as other tick species, may secrete various isoforms of the same protein and/or different members of the same family (but with similar functions) into saliva during blood feeding as a mechanism of antigenic variation to avoid recognition by the host's immune system.

Notably, almost all CDSs belonging to tick immune system were upregulated by feeding in SG of *A. sculptum*, excepted for lectin encoding sequences. Transcripts of tick immune system components, especially AMPs, were previously identified in tick sialotranscriptomes (Francischetti et al., 2009; Kotsyfakis et al., 2015a). AMPs secreted in tick saliva may prevent the growth of microbes at the feeding site as well as in tick gut (Karim and Ribeiro, 2015). Interestingly, the CDSs Acaj-65746, which encode a defensin, was highly induced in SG of ticks fed for 72 h. MSA and phylogenetic analysis showed that this AMP is member of the arthropod defensin family, also named as Knottin scorpion toxin-like in InterPro database (Gracy et al., 2008). One peptidoglycan recognition protein (PGRP) (AcajSIGP-81204) with amidase catalytic site was also upregulated in SG of *A. sculptum* by feeding. PGRPs are classified into non-catalytic or catalytic depending on the presence of the amidase catalytic site. While non-catalytic PGRPs function as pathogen pattern recognition receptors and activate immune pathways upon infection, catalytic PGRPs cleaves peptidoglycan, acting as effectors and/or negative regulators of the immune response (Palmer and Jiggins, 2015).

Members of the Da-p36 immunosuppressant family, putatively enrolled in host immunomodulatory activity, were also identified in *A. sculptum* SG and all of them were upregulated by feeding. Da-p36 was originally identified in both saliva and SG of *D. andersoni* and it presents an inhibitory activity on concanavalin A-induced proliferation of murine splenocytes (Bergman et al., 2000). Homologues of Da-p36 have been reported in others tick species, such as *Amblyomma variegatum* (Nene et al., 2002), *A. maculatum* (Karim et al., 2011), *Haemaphysalis longicornis* (Konnai et al., 2009), and *Rhipicephalus appendiculatus* (Nene et al., 2004). Evasins, small proteins that recognize and bind chemokines, were first described in *R. sanguineus* (Frauenschuh et al., 2007) and were also detected in *A. sculptum* sialotranscriptome. Evasin-1 and Evasin-4 binds CC chemokines, while Evasin-3 binds CXC chemokines, and Evasin-2 has no ligand characterized to date (Frauenschuh et al., 2007; Déruaz et al., 2008). An Evasin-3-like activity was also observed in SG extracts of adult *A. variegatum, R. appendiculatus*, and *Dermacentor reticulatus* (Vancová et al., 2010).

The 8.9 kDa superfamily is exclusively found in hard ticks, but none of its members were functionally characterized so far (Francischetti et al., 2009; Karim et al., 2011). Two members of this family are highly expressed in hemocytes of *I. ricinus* (Kotsyfakis et al., 2015a). Therefore, the authors suggested that they might be involved with tick immunity (Kotsyfakis et al., 2015a). Twenty three from 24 CDSs of proteins of this family were upregulated by blood feeding in tick SG. Glycine-rich proteins are members of another family of proteins specifically find in ticks. CDSs of glycine-rich proteins were also identified in *A. sculptum* sialotranscriptome. Glycine-rich proteins of ticks are associated to salivary cement used to attach mouthparts to host skin (Francischetti et al., 2009; Maruyama et al., 2010).

A blastp search of the sialotranscriptome of *A. sculptum* (formely named *A. cajennense*) against a collection of protein sequences of *A. cajennense* (Garcia et al., 2014; the “cajennense protein database;” please see column BG in Supplementary Table 1) was performed. About 25% of the 9,560 CDSs identified in the current study presented no match against the “cajennense database.” As the genome of *A. sculptum* is not available, this study not only provides additional evidences on the transcriptional changes stimulated by blood feeding in ticks, but also extensively contributed with novel transcripts for public sequence databases of this species.

To identify the proteins that are effectively secreted by *A. sculptum* SG, the proteome of the saliva of fed females was determined. A set of 124 proteins was identified, among which 58 are predicted to be secreted, reinforcing the importance of secreted proteins during the feeding process. Among secreted proteins, we highlight lipocalins, 8.9 kDa, glycine-rich proteins and microplusin-like AMPs. It was previously shown that the microplusin of *R. microplus* (Fogaça et al., 2004) exhibits the properties of chelating metallic ions, which seems to be involved in its activity against the Gram-positive bacterium *Micrococcus luteus* (Silva et al., 2009) and the fungus *Cryptococcus neoformans* (Silva et al., 2011). As mentioned above, the presence of proteins with antimicrobial properties in tick saliva might play a role in preventing the growth of microbes ingested with the blood meal (Karim and Ribeiro, 2015). Six vitellogenins were also detected in saliva of fed ticks. Vitellogenin is the major yolk precursor protein, being incorporated in eggs as vitellin (Taylor et al., 1991; Rosell and Coons, 1992; Chinzei and Yano, 1994; James et al., 1999; Thompson et al., 2007). The heme-binding property of both vitellogenin (Thompson et al., 2007) and vitellin (Logullo et al., 2002) has been previously shown. As ticks do not synthesize heme (Braz et al., 1999), these proteins are an important source of this prosthetic group for embryos development (Logullo et al., 2002). It was also demonstrated that vitellins also exhibit an antioxidant property, diminishing the heme-induced lipid peroxidation (Logullo et al., 2002). Importantly, *R. microplus* ticks fed on sheep vaccinated with vitellin showed reduced engorgement and oviposition rates (Tellam et al., 2002).

In conclusion, the current study shows that blood feeding exert a strong effect on the gene expression profile of the SG of *A. sculptum*, upregulating the transcription of putative secreted proteins, which may play pivotal role during the feeding process. In addition, this is the first report on the proteome of *A. sculptum* saliva. Transcriptional and protein data presented in this study amplify the knowledge of proteins possibly involved in tick feeding, which might also play a role on transmission of pathogens. Future functional studies to determine the role of such proteins on *A. sculptum* physiology as well as on transmission of *R. rickettsii* to the vertebrate host are warranted and might have potential as vaccine targets and as pharmacological bioproducts with significant biological activities.

## Author contributions

Designed the experiments: EE, AS-N, and ACF. Generated biological samples: EE, LAM, and FBC. Performed the experiments: EE and AAR. Analyzed data: JMCR, SRM, EE, ACF, and RK. Performed statistic data analysis: AF, JMCR, and SRM. Contributed reagents/materials/analysis tools: AS-N, MBL, JMCR, GP, and ACF. Wrote the paper: EE, SRM, AS-N, and ACF. All authors read and approved the final manuscript.

### Conflict of interest statement

The authors declare that the research was conducted in the absence of any commercial or financial relationships that could be construed as a potential conflict of interest.
