# Supplementary material for: Analysis of the Salivary Gland Transcriptome of Unfed and Partially Fed Amblyomma sculptum Ticks and Descriptive Proteome of the Saliva
Source: Front Cell Infect Microbiol. 2017 Nov 21;7:476. doi: 10.3389/fcimb.2017.00476 (PMC5702332; doi:10.3389/fcimb.2017.00476)
Supplement: Supplementary file 1 [file Table1.DOCX]

Supplementary Table 1

Please access the link below to download the complete dataset:

<http://exon.niaid.nih.gov/transcriptome/Amb_sculptum/Supplementary_Table_1-Web.xlsx>
