# Supplementary material for: Analysis of the Salivary Gland Transcriptome of Unfed and Partially Fed Amblyomma sculptum Ticks and Descriptive Proteome of the Saliva
Source: Front Cell Infect Microbiol. 2017 Nov 21;7:476. doi: 10.3389/fcimb.2017.00476 (PMC5702332; doi:10.3389/fcimb.2017.00476)
Supplement: Supplementary file 2 [file Table2.DOCX]

**Supplementary Table 2.** Accession numbers of proteins sequences used in multiple sequence alignment and phylogenetic analyses displayed in Figure 3, 4, 5 and 6.

| **Species of tick** | **Accession number** |
| --- | --- |
| ***Trypsin inhibitor-like (TIL) domain* (Figure 3)** | |
| *Amblyomma americanum* | JAG92207.1 |
| *Amblyomma cajennense* | JAC23686.1 |
| *Amblyomma cajennense* | JAC23917.1 |
| *Amblyomma maculatum* | AEO36723.1 |
| *Amblyomma parvum* | JAC26596.1 |
| *Antricola delacruzi* | AFI98404.1 |
| *Haemaphysalis longicornis* | BAE02553.1 |
| *Hyalomma excavatum* | JAP66368.1 |
| *Ixodes ricinus* | JAB78908.1 |
| *Ixodes scapularis* | XP_002399670.1 |
| *Ixodes scapularis* | XP_002410314.1 |
| *Ornithodoros brasiliensis* | JAT78789.1 |
| *Ornithodoros coriaceus* | ACB70395.1 |
| *Rhipicephalus appendiculatus* | JAP81603.1 |
| *Rhipicephalus haemaphysaloides* | AJS14621.1 |
| *Rhipicephalus pulchellus* | JAA54152.1 |
| *Sarcoptes scabiei* | KPM02579.1 |
| ***Kunitz domain* (Figure 4)** | |
| *Amblyomma cajennense* | [JAC19051.1](https://www.ncbi.nlm.nih.gov/protein/604793212?report=genbank&log$=prottop&blast_rank=2&RID=PU19E94R015) |
| *Amblyomma americanum* | [ACG76172.1](https://www.ncbi.nlm.nih.gov/protein/196476608?report=genbank&log$=prottop&blast_rank=9&RID=PU19E94R015) |
| *Amblyomma parvum* | [JAC26710.1](https://www.ncbi.nlm.nih.gov/protein/604808737?report=genbank&log$=prottop&blast_rank=11&RID=PU19E94R015) |
| *Rhipicephalus pulchellus* | [JAA62096.1](https://www.ncbi.nlm.nih.gov/protein/427793289?report=genbank&log$=prottop&blast_rank=14&RID=PU19E94R015) |
| *Amblyomma aureolatum* | [JAT93249.1](https://www.ncbi.nlm.nih.gov/protein/1073700385?report=genbank&log$=prottop&blast_rank=16&RID=PU19E94R015) |
| *Ixodes ricinus* | [JAB77957.1](https://www.ncbi.nlm.nih.gov/protein/556069881?report=genbank&log$=prottop&blast_rank=48&RID=PU19E94R015) |
| *Ornithodoros moubata* | [JAV99654.1](https://www.ncbi.nlm.nih.gov/protein/1202282436?report=genbank&log$=prottop&blast_rank=58&RID=PU19E94R015) |
| *Amblyomma triste* | [JAC30781.1](https://www.ncbi.nlm.nih.gov/protein/604816883?report=genbank&log$=prottop&blast_rank=23&RID=PU19E94R015) |
| *Parasteatoda tepidariorum* | [LAA08604.1](https://www.ncbi.nlm.nih.gov/protein/961309651?report=genbank&log$=prottop&blast_rank=9&RID=PZPX68HP016) |
|  |  |
| ***Defensin* (Figure 5)** | |
| *Carios mimon* | JAR87235.1 |
| *Carios puertoricensis* | ACJ04429.1 |
| *Ornithodoros moubata* | BAC10303.1 |
| *Rhipicephalus pulchellus* | JAA54212.1 |
| *Rhipicephalus appendiculatus* | JAP87934.1 |
| *Rhipicephalus sanguineus* | ACX53993.1 |
| *Hyalomma excavatum* | JAP67693.1 |
| *Ixodes ricinus* | JAB77866.1 |
| *Ixodes persulcatus* | BAH09304.1 |
| *Amblyomma americanum* | JAG92207.1 |
| *Amblyomma cajennense* | JAC22454.1 |
| *Amblyomma parvum* | JAC24893.1 |
| *Amblyomma triste* | JAC30338.1 |
| *Androctonus bicolor* | AIX87626.1 |
| ***Microplusin* (Figure 6)** | |
| *Amblyomma cajennense* | JAC23772.1 |
| *Amblyomma americanum* | JAG91859.1 |
| *Amblyomma triste* | JAC30433.1 |
| [*Rhipicephalus pulchellus*](https://blast.ncbi.nlm.nih.gov/Blast.cgi#alnHdr_427776963) | JAA53933.1 |
| *Rhipicephalus appendiculatu* | JAP82083.1 |
| *Hyalomma excavatum* | JAP63708.1 |
| *Amblyomma aureolatum* | JAT91325.1 |
| *Amblyomma parvum* | AC26578.1 |
| *Amblyomma maculatum* | AEO36119.1 |
| [*Rhipicephalus microplus*](https://blast.ncbi.nlm.nih.gov/Blast.cgi#alnHdr_629510454) | [JAC59074.1](https://www.ncbi.nlm.nih.gov/protein/629510452?report=genbank&log$=prottop&blast_rank=30&RID=PTXTCJWM014) |
