# Supplementary material for: Analysis of the Salivary Gland Transcriptome of Unfed and Partially Fed Amblyomma sculptum Ticks and Descriptive Proteome of the Saliva
Source: Front Cell Infect Microbiol. 2017 Nov 21;7:476. doi: 10.3389/fcimb.2017.00476 (PMC5702332; doi:10.3389/fcimb.2017.00476)
Supplement: Supplementary file 3 [file Table3.DOCX]

| **Primer name** | **Sequence 5' → 3'** | |
| --- | --- | --- |
|  | **Forward** | **Reverse** |
| ACAJ-77950 | AGTCAGGGACACCAGCAAAC | AAAATATCTTGCGCTGGCAC |
| ACAJ-56179 | CAAGAAATGCGTAAGCAGCA | GGCAACGGTTCCATTTAACA |
| ACAJ-73764 | TTCCTCGTTGTCTGCCTTCT | CCTCCGATGCTGTAGGTGAC |
| ACAJSIGP-81204 | TTCTCGAAAAAGAAGCCCAA | TAGCGAGGCCACTTCTTGAT |
| ACAJ-74654 | TCAGGTTTCGGTTTGGGTAG | CTAACTCTTCCTTGCCGTCG |
| ACAJ-65746 | GAGGAGAAGAGCGAGGAACC | CAGTTCGTACTTGCAGTGCC |
| ACAJ-81474 | CAAGGACGAGAAGGTCGAAG | TACTCCACCTACGCCAGGAC |
| ACAJ-81475 | TCTCGGTTACGGAGGTCTTG | AGCTGTAGCCCTGCTGGTTA |
| ACAJ-72892 | GCTCTACGACCAGGAGATCG | TCCTTCATCCGAGCAGTCTT |
| ACAJSIGP-29822 | TGAGACTCAGGTTGTGGCTG | TAACGGCCTTGAAGTTGTCC |
| ACAJSIGP-72252 | ACCCAAAGATCGTGTTCCTG | TCCAGTCTGCGAGTATGTCG |
| S3A (reference gene) | GATGGCTACCTTCTGCGAAT | TTTCCTGATGAGACGAACCT |

**Supplementary Table 3**. List of RT-qPCR primers used in this study.
