# Supplementary material for: Analysis of the Salivary Gland Transcriptome of Unfed and Partially Fed Amblyomma sculptum Ticks and Descriptive Proteome of the Saliva
Source: Front Cell Infect Microbiol. 2017 Nov 21;7:476. doi: 10.3389/fcimb.2017.00476 (PMC5702332; doi:10.3389/fcimb.2017.00476)
Supplement: Supplementary file 4 [file Table4.DOCX]

**Supplementary Table 4.** Proteins detected in saliva of fed *A. sculptum* ticks by LC-MS/MS and modulation of corresponding CDSs by RNA-seq.

| **CDSs corresponding to identified proteins** | **Modulation of CDSs**  **(fold-change in RNA-seq analysis)** | **Annotation** | **Number of biological replicates in which the protein was detected** | **Funcional class** |
| --- | --- | --- | --- | --- |
| ACAJTE-74903 | Downregulated (0,33) | Myosin class II heavy chain - probable fragment | 3 | *Cytoskeletal* |
| ACAJTE-80817 | Not modulated | Myosin class II heavy chain - probable fragment | 3 |  |
| ACAJ-71517 | Not modulated | Tumor-specific antigen (contains WD repeats) - probable fragment | 1 |  |
| ACAJ-71992 | Not modulated | Tropomyosin - probable fragment | 3 |  |
| ACAJ-72407 | Not modulated | Ca2+-binding actin-bundling protein (spectrin) alpha chain (EF-Hand protein superfamily) - probable fragment | 3 |  |
| ACAJ-72613 | Not modulated | The tubulin superfamily includes five distinct families | 3 |  |
| ACAJ-73522 | Downregulated (0,47) | Muscle-specific protein 20-like isoform 2 | 3 |  |
| ACAJ-75451 | Upregulated (2,26) | Myosin alkali light chain protein | 1 |  |
| ACAJ-76006 | Upregulated (2,70) | Calponin | 3 |  |
| ACAJ-76111 | Not modulated | Radixin moesin | 1 |  |
| ACAJ-76319 | Not modulated | Profilin binds actin monomers - 2 predicted membrane helices | 1 |  |
| ACAJ-76809 | Not modulated | Amb caj-87 similar to muscle lim protein isoform a isoform 1 amblyomma cajennense | 1 |  |
| ACAJ-78907 | Not modulated | Actin-2 - probable fragment | 3 |  |
| ACAJ-79247 | Not modulated | Adenylate cyclase-associated protein (CAP/Srv2p) | 1 |  |
| ACAJ-80255 | Not modulated | Actin depolymerizing factor | 3 |  |
| ACAJ-83327 | Not modulated | Troponin T skeletal muscle-like isoform X5 | 2 |  |
| ACAJ-85579 | Upregulated (3,42) | Alpha tubulin | 3 |  |
| ACAJTE-73768 | Not modulated | Myosin class II heavy chain - probable fragment | 1 |  |
| ACAJ-78167 | Upregulated (2,98) | Glutathione S-transferase mu class | 5 | *Detoxification* |
| ACAJ-64767 | Upregulated (9,25) | Reeler partial | 2 | *Extracellular matrix and adhesion* |
| ACAJ-73989 | Upregulated (4,00) | Nidogen - probable fragment | 1 |  |
| ACAJ-75348 | Upregulated (137,10) | Cuticle protein - signalp detected - 6 predicted membrane helices | 1 |  |
| ACAJ-76542 | Upregulated (26,68) | Structural constituent of cuticle partial - probable fragment - 24 netoglyc sites | 3 |  |
| ACAJ-81446 | Not modulated | Chitin binding peritrophin-a domain protein partial | 2 |  |
| ACAJ-85740 | Not modulated | Extracellular matrix glycoprotein Laminin subunit beta - probable fragment | 1 |  |
| ACAJSIGP-72696 | Downregulated (0,25) | Nidogen - signalp detected | 2 |  |
| ACAJSIGP-82833 | Upregulated (3,50) | Matricellular protein Osteonectin/SPARC/BM-40 - signalp detected | 2 |  |
| ACAJ-49265 | Upregulated (3,78) | Toll-like receptor 5 | 5 | *Immunity* |
| ACAJSIGP-74221 | Not modulated | Alpha-macroglobulin - probable fragment - signalp detected | 5 |  |
| ACAJ-77502 | Not modulated | Amb caj-54 histone 2a amblyomma cajennense | 3 | *Carbohydrate metabolism* |
| ACAJ-72976 | Upregulated (8,09) | Beta-N-acetylhexosaminidase - probable fragment | 1 |  |
| ACAJSIGP-85904 | Not modulated | Glucosidase II catalytic (alpha) subunit - signalp detected | 5 |  |
| ACAJSIGP-74712 | Upregulated (4,04) | Aldehyde dehydrogenase - probable fragment | 1 | *Energy metabolism* |
| ACAJSIGP-73942 | Upregulated (4,66) | Biotinidase and vanin - signalp detected | 2 | *Intermediary metabolism* |
| ACAJ-4378 | Upregulated (2,58) | Acyl-coa-binding protein | 2 | *Lipid metabolism* |
| ACAJ-74429 | Not modulated | Histone H4 | 4 | *Nuclear regulation* |
| ACAJ-79912 | Not modulated | PDZ domain found in a variety of Eumetazoan signaling molecules | 1 | *Protein export machinery* |
| ACAJ-56360 | Not modulated | Cathepsin B precursor | 1 | *Protein modification machinery* |
| ACAJ-56793 | Upregulated (11,69) | Chymotrypsin-elastase inhibitor ixodidin | 3 |  |
| ACAJ-67891 | Upregulated (3,42) | Lysosomal acid phosphatase | 5 |  |
| ACAJ-72045 | Not modulated | Multifunctional chaperone | 3 |  |
| ACAJ-72965 | Not modulated | 14-3-3 protein zeta multifunctional 14-3-3 family chaperone | 4 |  |
| ACAJ-73714 | Downregulated (0,23) | Heat shock protein partial - probable fragment | 2 |  |
| ACAJTE-74253 | Not modulated | Heat shock protein | 3 |  |
| ACAJSIGP-75574 | Upregulated (4,36) | Calreticulin - signalp detected | 3 |  |
| ACAJ-76861 | Not modulated | Midgut cysteine proteinase - probable fragment | 5 |  |
| ACAJ-77698 | Not modulated | Protein disulfide isomerase (prolyl 4-hydroxylase beta subunit) - signalp detected | 2 |  |
| ACAJ-79572 | Downregulated (0,23) | Heat shock protein - 15 netoglyc sites | 2 |  |
| ACAJSIGP-71621 | Not modulated | Heat shock protein 90 - signalp detected | 1 |  |
| ACAJSIGP-76333 | Upregulated (3,78) | Thioredoxin/protein disulfide isomerase - signalp detected | 2 |  |
| ACAJSIGP-77638 | Upregulated (7,84) | Protein disulfide isomerase (prolyl 4-hydroxylase beta subunit) - signalp detected | 3 |  |
| ACAJ-63857 | Not modulated | 40S ribosomal protein S28 | 1 | *Protein synthesis* |
| ACAJ-74577 | Not modulated | Elongation factor 1-gamma-like | 1 |  |
| ACAJ-69685 | Not modulated | Metastriate insulin growth factor binding protein | 4 | *Signal transduction* |
| ACAJ-71663 | Downregulated (0,44) | Calmodulin | 1 |  |
| ACAJ-72245 | Not modulated | Insulin-like growth factor binding protein-related protein 1 - signalp detected | 3 |  |
| ACAJ-73603 | Not modulated | G protein beta subunit-like protein | 1 |  |
| ACAJ-75503 | Not modulated | GDP dissociation inhibitor | 1 |  |
| ACAJ-60792 | Downregulated (0,008) | Vitellogenin-2 - probable fragment | 4 | *Storage* |
| ACAJ-72186 | Downregulated (0,32) | Lipoprotein amino terminal region | 3 |  |
| ACAJ-72735 | Not modulated | Apolipophorin | 3 |  |
| ACAJSIGP-72917 | Not modulated | Vitellogenin-B - signalp detected | 3 |  |
| ACAJSIGP-73526 | Upregulated (188,63) | Vitellogenin-2 - signalp detected | 5 |  |
| ACAJSIGP-76406 | Downregulated (0,14) | Vitellogenin-1 - signalp detected | 1 |  |
| ACAJSIGP-75024 | Upregulated (15,70) | Vitellogenin-2 - signalp detected | 5 |  |
| ACAJSIGP-76406 | Downregulated (0,14) | Vitellogenin-1 - signalp detected | 3 |  |
| ACAJ-33969 | Upregulated (818,57) | 8.9kDa_superfamily_35 8.9 kda family | 2 | *Putative secreted* |
| ACAJ-45572 | Upregulated (2,24) | Secreted peptide precursor - probable fragment | 5 |  |
| ACAJ-49256 | Upregulated (2,56) | Secreted peptide precursor - signalp detected | 5 |  |
| ACAJ-57400 | Not modulated | Microplusin_1 | 1 |  |
| ACAJ-63852 | Not modulated | Serine proteinase inhibitor precursor - signalp detected | 1 |  |
| ACAJ-67443 | Upregulated (4,55) | Secreted salivary gland peptide - signalp detected | 2 |  |
| ACAJ-68144 | Upregulated (569,91) | 8.9kda_1 - signalp detected 8.9 kda family | 1 |  |
| ACAJ-73258 | Upregulated (852,85) | Hypothetical secreted protein 94 - signalp detected | 2 |  |
| ACAJ-77327 | Upregulated (6,64) | Conserved secreted protein precursor - signalp detected | 1 |  |
| ACAJ-77327 | Not modulated | Conserved secreted protein precursor - signalp detected | 1 |  |
| ACAJ-77500 | Upregulated (5,58) | Microplusin-like antibacteral peptide - signalp detected | 5 |  |
| ACAJ-74161 | Upregulated (4,69) | 8.9kda_1 - signalp detected 8.9 kda family | 5 |  |
| ACAJ-81474 | Downregulated (0,20) | Glycine-rich cell wall structural protein - signalp detected | 1 |  |
| ACAJ-81475 | Downregulated (0,24) | Glycine-rich cell wall structural protein - signalp detected | 1 |  |
| ACAJ-81582 | Downregulated (0,49) | Secreted salivary gland peptide - signalp detected | 5 |  |
| ACAJ-76805 | Upregulated (8,25) | Lipocalin-3_1 lipocalin | 1 |  |
| ACAJ-76856 | Upregulated (586,90) | Lipocal-1_14 lipocalin | 1 |  |
| ACAJ-79229 | Upregulated (2,25) | Cystatin - signalp detected | 4 |  |
| ACAJSIGP-12678 | Upregulated (5,78) | Tick salivary thyropin - signalp detected | 5 |  |
| ACAJSIGP-14644 | Upregulated (3,32) | Lipocalin-2_1 - signalp detected lipocalin | 5 |  |
| ACAJSIGP-14784 | Upregulated (4,63) | Microplusin-like antibacteral peptide - signalp detected | 1 |  |
| ACAJSIGP-17283 | Upregulated (3,96) | 8.9kda_1 - signalp detected 8.9 kda family | 1 |  |
| ACAJSIGP-22470 | Upregulated (2,14) | Conserved secreted protein precursor - signalp detected | 1 |  |
| ACAJSIGP-26045 | Upregulated (2,15) | Conserved secreted protein precursor - signalp detected | 4 |  |
| ACAJSIGP-27978 | Upregulated (7,62) | Hypothetical secreted protein precursor | 1 |  |
| ACAJSIGP-28297 | Upregulated (4,44) | Hypothetical secreted protein 790 - signalp detected | 3 |  |
| ACAJSIGP-26045 | Upregulated (2,15) | Conserved secreted protein precursor - signalp detected | 1 |  |
| ACAJSIGP-30590 | Upregulated (13,83) | Secreted protein - signalp detected | 5 |  |
| ACAJSIGP-32934 | Upregulated (5,97) | Lipocalin-2_25 - signalP detected lipocalin | 3 |  |
| ACAJSIGP-33818 | Upregulated (65,26) | Hypothetical secreted protein precursor | 1 |  |
| ACAJSIGP-34265 | Upregulated (2,54) | Hypothetical secreted protein precursor | 1 |  |
| ACAJSIGP-48314 | Not modulated | Thyropin precursor - signalp detected | 4 |  |
| ACAJSIGP-4975 | Not modulated | Endonuclease partial - probable fragment - signalp detected | 2 |  |
| ACAJSIGP-5845 | Upregulated (3100,33) | Serine proteinase inhibitor - signalp detected | 1 |  |
| ACAJSIGP-60021 | Not modulated | Conserved secreted protein precursor - signalp detected | 5 |  |
| ACAJSIGP-68214 | Upregulated (15392,82) | U3-aranetoxin-ce1a - signalp detected | 1 |  |
| ACAJSIGP-74350 | Not modulated | Conserved secreted protein precursor - signalp detected | 1 |  |
| ACAJSIGP-74741 | Upregulated (25,08) | Lipocalin-2_28 - signalp detected lipocalin | 1 |  |
| ACAJSIGP-74586 | Not modulated | Serine carboxypeptidase - signalp detected | 1 |  |
| ACAJSIGP-75254 | Upregulated (7,28) | Cathepsin L-like cysteine proteinase B - signalp detected | 5 |  |
| ACAJSIGP-75415 | Upregulated (3,04) | Hypothetical secreted protein precursor | 3 |  |
| ACAJSIGP-75535 | Downregulated (0,43) | Cathepsin C precursor - signalp detected | 2 |  |
| ACAJSIGP-76888 | Upregulated (3,91) | Conserved secreted protein precursor - signalp detected | 2 |  |
| ACAJSIGP-79331 | Downregulated (0,24) | Lipocalin-1_16 - signalp detected lipocalin | 1 |  |
| ACAJSIGP-80410 | Upregulated (4,77) | Hypothetical secreted protein 790 - signalp detected | 5 |  |
| ACAJSIGP-80555 | Upregulated (5,69) | Conserved secreted protein precursor - signalp detected | 1 |  |
| ACAJSIGP-81529 | Not modulated | Aspartic protease - signalp detected | 1 |  |
| ACAJSIGP-81986 | Upregulated (2,80) | Prolylcarboxypeptidase - signalp detected | 1 |  |
| ACAJSIGP-83508 | Upregulated (17,19) | Hypothetical secreted protein precursor lipocalin | 2 |  |
| ACAJSIGP-83683 | Downregulated (0,46) | Deoxyribonuclease II partial - probable fragment - signalp detected | 3 |  |
| ACAJSIGP-85320 | Upregulated (5,68) | Tick_mucins_1 - signalp detected | 1 |  |
| ACAJSIGP-75579 | Not modulated | Cystatin - signalp detected | 5 |  |
| ACAJSIGP-75698 | Not modulated | BT_66 - signalp detected- Basic tail protein | 2 |  |
| ACAJSIGP-76090 | Not modulated | Secreted metalloprotease | 2 |  |
| ACAJ-57394 | Upregulated (27,57) | Hypothetical glycine-rich secreted cement protein - 4 predicted membrane helices - Conserved membrane protein | 4 |  |
| ACAJSIGP-9558 | Not modulated | Salivary secreted protein - signalp detected | 4 |  |
| ACAJ-803 | Upregulated (3,63) | 5'-nucleotidase | 1 |  |
| ACAJ-77165 | Not modulated | Unknown product partial | 5 | *Viral products* |
